# Treatment Outcomes and Prognostic Factors in Mexican Patients with Endometrial Carcinoma with Emphasis on Patients Receiving Radiotherapy after Surgery: An Institutional Perspective

**DOI:** 10.5402/2012/178051

**Published:** 2012-05-17

**Authors:** Christian Flores, Carlos Mariscal, Alfredo Celis, Nidia M. Balcázar, Abelardo Meneses, Alejandro Mohar, Aida Mota, Elizabeth Trejo

**Affiliations:** Department of Radiation Oncology, National Cancer Institute, St. Fernando's Avenue 22, Tlalpan, Mexico City, Mexico

## Abstract

*Aim*. To analyze the clinical characteristics and treatment outcomes in patients with endometrial carcinoma treated in a Latin American institute with emphasis in patients receiving adjuvant radiotherapy. *Methods*. A total of 412 patients with endometrial carcinoma admitted to our hospital between 1998 and 2008 were evaluated, retrospectively. The mean age was 55 years (28–87). Two hundred seventy patients received RT following surgery. Stage distribution was as follows: 221 patients (54%) stage I, 86 patients (21%) stage II, and 103 patients (24.5%) stage III and 2 patients (0.5%) stage IVA. *Results*. Overall survival rate was 95% at 2 years, 84% at 5 years, and 79% at 10 years. By the end of followup, 338 patients (82%) were disease-free, and 13 (3%) were alive with disease. Univariate and multivariate analyses identified age, grade, serosal and adnexial involvement as significant predictors for overall survival. *Conclusion*. The results of our study suggests that early-stage, low-grade endometrial cancer with no risk factors should not receive external beam radiotherapy, intermediate risk patients should receive only vaginal vault brachytherapy, and the use of chemotherapy with radiotherapy for patients high-risk and advanced-stage carcinoma the addition of radiotherapy is associated with a better survival being an effective therapeutic option.

## 1. Introduction

Endometrial carcinoma is one of the most common gynecologic malignancies in Mexican women after uterine cervix, ovarian and breast cancers [1]. In our country, cancer has had an important rise in the last 50 years. According to the last data from National Tumor Registry in year 2003, a total of 71,901 new cancer cases in women were registered, from these cases, 1552 belonged to uterine corpus carcinoma [2]. During years 2000–2004 in our hospital 19,264 new cases were reported with 64.6% (12,444) belonged to women. Specifically uterine corpus carcinoma ranked the fifth place for gynecologic tumors being endometrioid adenocarcinoma the most prevalent histology [3].

 Fortunately, 70% of endometrial cancer patients are diagnosed with localized disease, resulting in a 5-year survival of 95% in this subset of patients [4, 5]. The median age at diagnosis is 62 years [6]. Risk factors for development of this disease include obesity, diabetes, hypertension, endogenous or exogenous estrogen, nulliparity, menopause, family history, and endometrial hyperplasia. In addition, with better surgical techniques, advances in radiotherapy and chemotherapy, the mortality rate for endometrial cancer continues to decline.

 In Mexico, most endometrial cancers are surgically staged according to criteria established by the International Federation of Obstetrics and Gynecology (FIGO), this includes an hysterectomy (TAH), bilateral salpingo-oophorectomy (BSO), and pelvic/para-aortic lymph node dissection (LND), with pelvic washings and an omental biopsy [7, 8]. Management and adjuvant treatment after surgery depends upon a patient's risk factor for recurrence, options include vaginal vault brachytherapy (VBT), pelvic external-beam radiation therapy (EBRT), and/or chemotherapy (CT). The most significant risk factor considered in any decision for adjuvant therapy include age, grade, histologic type, deep of myometrial invasion, tumor extension, tumor extension beyond the uterus, and lymphovascular space invasion. Depending of these risk factors, patients are grouped into low, intermediate or high risk for recurrence. Most controversy and debate is associated with the patients stratified to the intermediate-risk group.

 In this study, we analyzed the clinical characteristics and treatment outcomes in patients with endometrial cancer who received adjuvant radiotherapy after surgery. Although FIGO announced an update for endometrial cancer staging in October 2009 [9], all the cases reviewed in this study were staged according to FIGO 1988. 

## 2. Material and Methods 

 A total of 869 women were admitted at the National Cancer Institute in Mexico City between January 1998 and December 2008. Inclusion criteria included Mexican nationality, >18 years old at diagnosis, stages I to IVA and histologies: endometrioid, papillary cell, clear cell, and adenosquamous. Patients were excluded if they received EBRT previously or were diagnosed with metastatic disease, uterine sarcoma, not operated and treated with EBRT only, and the presence of another synchronic tumor (i.e., breast and endometrial carcinoma) with the exception of nonmelanoma skin cancer. Four Hundred and twelve women met the inclusion criteria and were evaluated retrospectively with regard to patient characteristics and treatment outcomes. All patients were assessed at the time of diagnosis with physical examination, routine blood counts and biochemical profile, chest X Rays, and pelvic tomography was performed in some women. All patients included in this study had negative pelvic or para aortic nodes on imaging. After systematic evaluation, 120 patients were treated with TAH-BSO, 20 patients with TAH-BSO-pelvic LND, and 271 patients with TAH-BSO-pelvic/para aortic LND and omentum biopsy. Histopathological and immunohistochemical assessments were performed in all cases. The staging was based on FIGO 1988 surgical staging system. A total of 270 women received adjuvant radiotherapy, EBRT alone was administered to 11 patients, 8 patients were administered total abdominal and pelvic EBRT plus VBT, 241 patients received EBRT + VBT, 7 patients were administered only VBT, and 7 patients received EBRT to retroperitoneum and pelvis besides VBT. External beam radiotherapy was given with cobalt units and 6 MV and 15 MV linear accelerators. A computed tomography scan with 5 mm cut was performed for all patients from the top of L4 to the bottom of lesser trochanters of the femurs as part of virtual simulation. Pelvic RT was administered with 4-field box method with a daily dose of 2 Gy to achieve a total dose of 50 Gy, in all cases, two-thirds of dose were administered through anterior and posterior fields, and one-third of the dose was administered through opposed lateral fields. For anterior and posterior fields (AP/PA) the upper limit was at the top of L5, lower field limit was at the bottom of the obturator foramina to cover vaginal cuff, lateral field limits were 2 cm beyond the bony margins of the pelvic inlet. Lateral opposed fields limits were the same as AP/PA fields in superior/inferior borders but in front of the pubis symphysis in anterior border and at S2-S3 level in posterior border. In patients whose para-aortic nodes were positive after surgery (Stage III), additional para aortic RT was administered at a total dose of 45 Gy with 1.8 Gy fractions, and patients with total abdominal radiotherapy were administered a total dose of 30 Gy en 1.5 Gy fractions trying to protect both kidneys and liver. Most patients received low-dose rate brachytherapy with 2, 3, and 3.5 cm cylinder applicator covering all vaginal length with a prescribed mean dose of 35 Gy at 0.5 cm depth. Doses for vagina, urinary bladder, and rectum were calculated in all patients to be under 80% of the defined doses. Side effects at vagina, urinary bladder, and rectum were classified according to the acute and late toxicity criteria as defined by the Radiation Therapy Oncology Group (RTOG) [10]. Clinical and radiological assessments were performed every 3 months for the first 2 years, every 6 months for the following 2 years, and annually thereafter. Radiographic surveillance was not routinely performed except for suspicion of disease recurrence or metastasis. Failures found on clinical or radiographic examination were classified as vaginal, local-regional (recurrence within the pelvis including vaginal cuff), or distant (bone, lung, liver, or otherwise). Deaths were recorded for analysis.

## 3. Statistical Methods 

The evaluated end point was overall survival (OS) using the Kaplan-Meier method, and significance was determined by the log-rank test. Univariate and multivariate analyses were performed using the Cox proportional hazards model. Statistical analyses were performed using statistical package for social sciences (SPSS) for windows version 17 (SPSS, Chicago, IL, USA).

## 4. Results

The mean age was 55 years (range: 23–87 years). At the time of diagnosis, 61 patients (14.8%) were premenopausal, and 351 (85.2%) were postmenopausal. Thirty-three percent of patients were obese (mean BMI 28.97 kg/m^2^), and 35.2% of women had a concomitant disease (diabetes mellitus, systemic arterial hypertension, or both). Clinical and pathological characteristics of patients are described in Table 1.

Three hundred fifty-nine patients (87.1%) had endometrioid cancer (see Table 2).

Histological grades were as follows: 165 (40%) low grade, 115 (27.9%) intermediate grade, and 130 (31.6%) high grade. Of the participants, 221 (53.6%) had myometrial (greater than half of the myometrial thickness), 57 (13.8%) had lymphovascular, 47 (11.4%) had serosal, and 38 (9.25%) had adnexial involvement. Twenty-seven patients (4.1%) had para aortic lymph node involvement, and 39 patients (9.5%) had pelvic lymph node involvement. FIGO stages were as follows: 221 patients (53.7%) stage I, 86 patients (20.9%) stage II, and 103 patients (24.9%) stage III, and 2 patients (0.5%) stage IVA. In Table 2, pathology characteristics of the 412 patients with endometrial carcinoma are described.

From these women, 211 (51.2%) were classified as high-risk recurrence group.  Table 3 describes the frequency of histological grade according each stage of disease. 

 After systematic evaluation, 120 patients were treated with TAH-BSO, 20 patients with TAH-BSO-pelvic LND, and 271 patients with TAH-BSO-Pelvic/para aortic LND and omentum biopsy. Histopathological and immunohistochemical assessments were performed in all cases. The staging was based on FIGO 1988 surgical staging system. A total of 270 women received adjuvant radiotherapy, EBRT alone was administered to 11 patients, 8 patients were administered whole abdominal and pelvic EBRT plus VBT, 241 patients received EBRT + VBT, 7 patients were administered only VBT and 7 patients received EBRT to retroperitoneum and pelvis besides VBT. External beam radiotherapy was given with cobalt units and 6 MV and 15 MV linear accelerators After surgery, radiotherapy started in a mean time of 84 days, and although available literature states starting adjuvant RT within first 6 weeks or after complete healing occurs, it is difficult to reach these policies at our institute because of the high demand of patients requiring radiotherapy and poor availability of linear accelerators. Sixty-three percent of patients had mild acute symptoms related to treatment, they were prescribed pharmacologic management with no interruptions because of severe adverse effects. Seventeen percent of patients experienced chronic symptoms, grade 2 radiation proctitis (RTOG) was the most common side effect. There were no women with acute grade 3-4 or late 3-4 gastrointestinal or genitourinary side effects. Adjuvant chemotherapy or hormone therapy used in patients was found to have adverse pathological factors such as affected nodes or positive peritoneal washings (see Table 4). 

**Figure 1:**
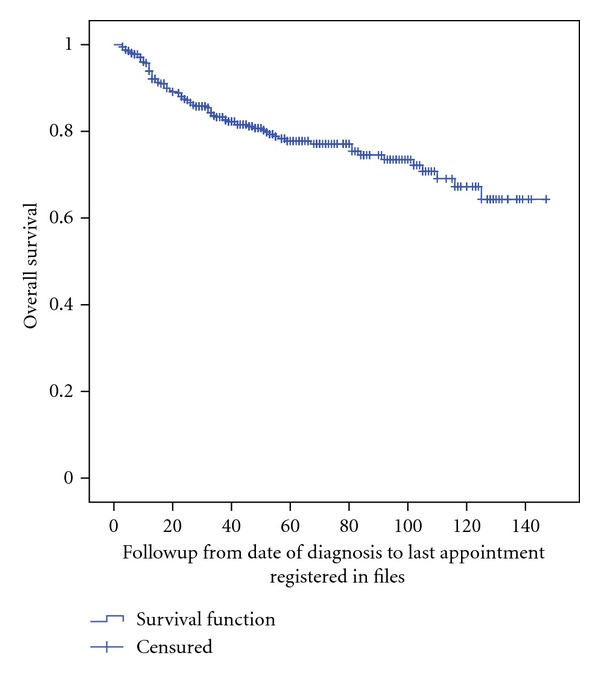
Overall survival of 412 patients with endometrial carcinoma.

Recurrences occurred in the first 2 years at a rate of 18% (7–63 months). Vaginal recurrence was reported in 15 patients, pelvic recurrence developed in 9, and distant metastasis in 27 patients being lung and liver the most common sites of recurrence. All patients with local or distant recurrence received systemic chemotherapy. After an average followup of 49 months, 338 patients (82%) were disease-free, and 13 (3.2%) were still alive with endometrial carcinoma. By the time this analysis was done, 85 patients were death (21%). Overall survival rate was 95% at 2 years, 84% at 5 years, and 79% at 10 years (Figure 1). 

 Univariate and multivariate analyses showed that age, histological grade, serosal and adnexial involvement were significantly associated with overall survival(*P* = 0.0003, *P* = 0.0004, *P* = 0.005, and *P* = 0.005, resp.) (Table 5).

## 5. Discussion

 Endometrial cancer is one of the most common gynecologic malignancies in Mexican women, and the most common in the United States. In 2010, it was estimated that 43,470 women were diagnosed with endometrial cancer (6% of new cancer cases), and 7950 women will die of the disease. The vast majority of women will fortunately survive their disease and be rendered cured given that approximately 75% of the patients are diagnosed with early-stage disease [11]. Usually the median age at diagnosis is 62 years [4, 5] and the majority of patients being postmenopausal, likewise, in the present study, the mean age of the subjects was 55 years, and 85% were postmenopausal. Hypertension, cardiac disease, and/or diabetes mellitus were present in 35% of the patients at the time of diagnosis. 

 The current treatment for endometrial carcinoma involves the use of surgery, radiation therapy, hormone therapy, and chemotherapy, either alone or sequentially. Definitive diagnosis requires histopathological examination, and staging is based on FIGO surgical staging system [12]. Surgery is the mainstay treatment for most patients with endometrial cancer. In a small subset of patients who present with unresectable disease or those whose disease is resectable but medically inoperable, definitive radiation is used instead as the primary treatment. The surgical management of endometrial cancer often involves simple hysterectomy and BSO with or without lymph node staging. However, pelvic and para aortic lymph nodal sampling and dissection has become a routine around the world, especially for patients operated by gynecologic oncologists [13–15], although this ultimate proceeding is controversial, two recent trials did not show improvement in disease-free or overall survival after lymphadenectomy in early-stage disease [16, 17]. Whether the procedure is being performed by a gynecologist or a gynecologic oncologist, the patient's general condition, the habitus of the patient, and several other factors affect the thoroughness of the lymphadenectomy. The minimum number of lymph nodes that need to be retrieved for the dissection to be representative has yet to be determined, and the final evaluation of a lymphadenectomy specimen will also depend on how meticulously the specimens are examined by the pathologist. Also, pelvic lymph nodal involvement is likely a marker for a biologically aggressive disease with a propensity for distant failures; any pelvic radiation offered in that setting is aimed at essentially improving local-regional control. However, the concern has been raised that omitting lymphadenectomy in patients with grade 1 tumors may lead to inappropriate postoperative treatment [18]. With regard to the type of surgery in our study 120 patients were treated with TAH-BSO, 20 patients with TAH-BSO-pelvic LND, and 271 patients with TAH-BSO-Pelvic/para-aortic LND and peritoneal washings and omentum biopsy. 

Approximately 70–80% of patients with endometrial cancer are diagnosed at stage I with a favorable prognosis [17, 19, 20]. Because the concept of stage grouping is based on creating groups with similar prognoses, it is not surprising that the outcome gets less favorable with advancing stage. Reported results of stage-based survival are remarkably consistent, with stages I, II, III, and IV disease having 5-year survival rates of 92%, 80%, 40%, and 5%, respectively [21]. In the present study, 221 (53.7%) of the patients had stage I disease at the time of diagnosis, while 86 patients (20.9%) had stage II, and 103 patients (24.9%) had stage III disease, and 2 patients (0.5%) had stage IVA disease. 

The low-risk group includes patients with stage IA G1, G2 tumors. The risk of pelvic lymph nodal positivity [22] is less than or equal to 3%, and the 5-year progression-free survival rate in this group is on the order of 90% to 96%. It is unlikely that postoperative pelvic external-beam radiation would add anything to the final outcome, and therefore radiation is not routinely recommended to this group of patients [23–26]. The role of intravaginal radiation in these patients is also of questionable benefit because of a very low rate of vaginal recurrence with surgery alone. These patients have the lowest risk of recurrence [27]. This was verified by the findings of prospective studies conducted between 1977 and 1983 by the Gynecologic Oncology Group (GOG 33), which investigated the patterns of failure for early-stage disease [22]. Of the women who had no myometrial invasion with grade 1 or 2 histology, none experienced recurrence [23]. In addition, a Cochrane review of the treatment for stage I endometrial cancer found a statistically significant greater risk for death in patients who had EBRT versus no treatment with low risk factors (i.e., disease confined to the endometrium, <50% myometrial invasion or grade 1/2) [17]. These data were confirmed again by meta-analysis of seven randomized trials by Johnson and Cornes showing that prophylactic EBRT could be harmful or ineffective in improving survival in women with low- or intermediate-risk cancer [19].

The intermediate-risk group has been defined by the GOG as those with pathologic stage IB to IIB (occult cervical involvement). It is in this risk category in which most of the controversy resides about the indication and type of radiation needed. With regard to the indication of adjuvant radiation in intermediate-risk patients, the controversy relates to the lack of improvement in overall survival with the use of adjuvant radiation compared with surgery alone. The two recently completed randomized trials demonstrating lack of survival advantage leads to the conclusion that adjuvant radiation is not needed. The policies for the use of radiotherapy have changed compared with trials, where EBRT + VBT were obligated for every patient operated with endometrial carcinoma [13, 14]. With more common use of lymphadenectomy during surgery, the value of adjuvant pelvic RT given to lymph-node negative areas has become questioned, and now patients from the intermediate risk group seem to benefit only from adjuvant VBT [15, 16, 28]. The inclusion criteria for this group vary slightly from trial to trial. These risk factors include, age, lymphovascular space invasion, tumor size, cervical involvement, and deep myometrial invasion. Despite multiple randomized studies, this group is still the most controversial with regard to adjuvant radiotherapy because it is not clear whether the benefit of treatment outweighs the risks. In a trial conducted by Aalders et al., all patients received vaginal brachytherapy and then were randomized to no further treatment or EBRT. There was a significant reduction in pelvic and vaginal recurrences in patients who received EBRT although these patients had more distant metastases. The 5-year overall survival (OS) was not improved by EBRT, although a more detailed analysis of the series concluded that patients with higher risk factors such as poorly differentiated tumors (grade 3), who have greater than 50% myometrial invasion might benefit from EBRT [29]. 

The Postoperative Radiation in Endometrial Cancer (PORTEC 1) trial in which 714 patients with stage IB G2,3 and IC G1,2 were randomized after TAH-BSO and without lymph nodal sampling to observation (*N* = 360) or pelvic radiation (*N* = 354) to a total dose of 46 Gy in 23 fractions. With a median followup of 52 months, the 5-year locoregional control was 96% in the radiation arm compared with 86% in the observation arm (*P* < 0.001). The corresponding 5-year survival rates were 81% and 85%, respectively (*P* = 0.37) [16]. Recently a PORTEC 1 fifteen year actualization confirmed the highly significant improvement of local control obtained by EBRT but an absence of survival benefit. Besides, a trend to develop second primary cancers was found over 15 years in 19% of all patients, 22% versus 16% for EBRT versus no additional treatment (*P* = 0.10), with observed versus expected ratios of 1.6 (EBRT) and 1.2 (no additional treatment) compared with a matched population (*P* = NS) [30]. Of the randomized studies, the GOG 99 study was the only study that required FIGO surgical staging. three hundred ninety-two patients with stage IB to IIB endometrial cancer who underwent TAH-BSO and pelvic and para aortic lymph nodal sampling were randomized to observation (*N* = 202) or postoperative pelvic radiation (*N* = 190) to a total dose of 50.4 Gy in 28 fractions. The study was designed to have an 80% chance of detecting a 58% decrease in the recurrence hazard rate and a 56% decrease in death hazard rate. The primary outcome was recurrence-free interval and is defined as the time from study entry to clinical, histologic, or radiographic evidence of disease recurrence. With a median followup of 69 months, the 2-year cumulative incidence of recurrence was 12% in the observation group and 3% in the adjuvant radiotherapy group. This was statistically significant (*P* = 0.007). The estimated 4-year survival rate was 86% in the observation group, compared with 92% in the irradiated group. This was not a statistically significant difference (*P* = 0.557) [31]. A recurrence rate of 12–14% was reported in patients treated with surgery alone in PORTEC and GOG-99 trials, with isolated vaginal recurrence as the most common form of recurrent disease [16, 31]. With regard to side effects, a complication rate of 25% and 15%, respectively, was reported for patients receiving pelvic RT in these two trials. The complication rate associated with the pelvic irradiation in the GOG-99 study was much higher than that in the PORTEC study, probably because of the extent of surgical staging. The rate of severe (grade 3 or 4) gastrointestinal toxicity with radiation were 8% in the GOG study but only 3% in the PORTEC trial. In our study, 17% of the patients had RTOG grade 1-2 late side effects mainly gastrointestinal effects, with 3 patients having grade 3-4 side effects. 

The ASTEC-EN5 trial, which is a pooled meta-analysis of two randomized trials (ASTEC and EN.5), was designed to evaluate the benefit of postoperative adjuvant EBRT in women with intermediate- and high-risk early-stage endometrial cancer. Between 1996 and 2005, 905 patients with intermediate-risk or high-risk endometrial cancer were randomly assigned to observation or external-beam radiotherapy. Vaginal brachytherapy was allowed based on each center's policy. As a result, 51% of the observation group received vaginal brachytherapy. Not surprisingly, the analysis did not find any evidence of overall survival benefit with external-beam radiotherapy [32]. 

The only trial comparing VBT versus RT is the second study in the PORTEC series. This noninferiority multicenter trial randomized patients with a high intermediate risk of recurrence defined as age 60 years and above and stage IC grades 1 or 2 or stage IB grade 3, and any age with stage IIA grades 1, 2 or grade 3 with less than 50% invasion. A total of 427 patients were recruited to this trial between 2002 and 2006, with a median follow-up time of 45 months. There was no significant difference in OS and the 5-year locoregional recurrence rate between treatment modalities. The lack of difference in survival in PORTEC-2 raises the question of whether adjuvant therapy is needed at all for patients with intermediate-risk disease. The estimated 5-year vaginal recurrence rates were 1.8% (95% CI) after VBT and 1.6% (95% CI) after EBRT [33]. Regarding the quality of life for patients enrolled in this study, the EBRT group reported significant and clinically relevant higher levels of diarrhea and fecal leakage, which limited social and daily activities for the EBRT group (*P* < 0.001) [34]. 

It is well known that pelvic RT helps to reduce locoregional recurrences and mortality in patients with multiple high risk factors (myometrial invasion greater than 50%, grade 3, gross involvement of the cervix or advanced-stage disease, lymphovascular space involvement, and aggressive histologic types) but with no benefit for the risk of distant metastases. In 2004, Creutzberg and colleagues [35] reported their findings for 104 patients with stage IC, grade 3 tumors who had registered but were not included in the first PORTEC trial, comparing their survival with that of the patients included in that trial. Grade 3 tumors were found to have a much higher metastasis rate than grades 1 or 2 tumors; multivariate analysis showed that histologic grade 3 was the most important adverse prognostic factor for relapse and death from endometrial cancer. These findings raise the question of whether radiation alone is enough for grade 3 tumors or whether the better, strategy may be a combination of radiation and chemotherapy. For such patients, chemotherapy (CT) has been proposed as a potentially beneficial therapeutic approach with randomized controlled trials specific to this exact subset of patients. Multiple studies have addressed which treatment might be better should they be combined, and if combined, what chemotherapy should be used. Still, the search for effective cytotoxic agents for the treatment of endometrial carcinoma continues. To date only three drugs with definite activity have been identified: doxorubicin, cisplatin, and paclitaxel [36]. 

Early results of a study done by the Nordic Society of Gynecologic Oncology and the European Organization for Research and Treatment of Cancer were presented at the American Society of Clinical Oncology meeting in May 2007 [37]. The NSGO-EC-9501/EORTC-55991 trial randomly assigned 382 patients between 1996 and 2007 to receive adjuvant radiation therapy (*n* = 196) or radiation therapy plus chemotherapy (*n* = 186). Patients with surgical stages I, II, IIIA (positive peritoneal fluid cytology only), or IIIC (positive pelvic lymph nodes only) disease were eligible if they were considered to be at sufficiently high risk for micrometastatic disease to qualify for adjuvant therapy. Patients with serous, clear cell, or anaplastic carcinomas were eligible regardless of risk factors. Patients with para aortic metastases were not eligible. Lymph node exploration at surgical staging was optional. All patients underwent at least a total abdominal hysterectomy with bilateral salpingo-oophorectomy. Pelvic radiation therapy, with or without vaginal brachytherapy, was given to a dose of 44 Gy. Chemotherapy was given before or after the radiation. Before August 2004, the chemotherapy regimen consisted of four courses of cisplatin and doxorubicin or epirubicin; after that time, several chemotherapy regimens were allowed. The median follow-up time was 4.3 years. The hazard ratio for progression-free survival in the study was 0.62 (95% CI, 0.40 to 0.97; *P* = 0.03), which translated to an estimated difference in 5-year progression-free survival rate of 7% (from 72% for those given radiation to 79% for those given chemoradiation therapy) [37]. The corresponding findings for overall survival were similar; the hazard ratio was 0.65 (95% CI, 0.40 to 1.06; *P* = .08), for an estimated difference in 5-year overall survival rate of 8% (74% for radiation versus 82% for chemoradiation therapy). The investigators concluded that even though 27% of the patients assigned to the chemoradiation therapy group received little or none of the prescribed chemotherapy, chemoradiation therapy was still better than radiation alone for patients with early endometrial cancer at high risk for micrometastasis [37]. The Japanese GOG was a randomized study that enrolled 475 patients with stages IC–IIIC endometrial carcinoma with deeper than 50% myometrial invasion (FIGO 1988 criteria). They were randomized to receive adjuvant EBRT or cyclophosphamide, doxorubicin, and cisplatin. A pelvic lymphadenectomy was performed in 96.1% of the patients and a para aortic lymphadenectomy was performed in 28.6%. The OS and PFS were not statistically different between the two arms. In their subgroup analysis of HIR patients, they found a significant benefit in PFS rate (*P* = 0.24) and OS rate (*P* = 0.006) for the chemotherapy group [38]. In the present study, 3 patients received CT alone, 25 concurrently with RT and 31 in sequential mode (CT first). 

For many years, adjuvant wide-field RT+CT has been used for advanced stage patients [39, 40]. However, GOG-122 study recruiting stages III-IV patients showed a survival advantage for CT alone compared to whole-abdominal RT [39]. Eight patients in our study received whole abdominal RT and during the postoperative period, among stage III patients, three received para aortic RT+BRT, and 241 received pelvic RT+BRT. 

The following risk factors were identified in studies evaluating the prognosis in endometrial cancer: age at the time of diagnosis, menopausal status, stage, tumor size, histological type, grade, depth of the myometrial invasion, cervical, stromal and lymphovascular invasion, adnexial involvement, intraperitoneal involvement, positive peritoneal cytology, and pelvic and para aortic lymph node involvement [25, 34, 41]. In our study, the significance of these factors was examined with univariate and multivariate analyses, showing statistical significance for overall survival for age, grade, and serosal and adnexial involvement (*P* = 0.05). 

The overall survival at 2, 5, and 10 years was 95%, 84%, and 79.4%, respectively, these results are consistent with others reported in the literature [28]. After a mean followup of 49 months, 338 patients (82%) were still alive without disease, 13 (3%) were alive with disease, and 85 (21%) were dead by the time this analysis was done. Recurrences presented in the first two years after treatment in 18% of patients being vaginal and locoregional (pelvic) the most common. 

## 6. Conclusion

This retrospective analysis of 412 women with endometrial cancer reveals statistically significant differences in pathologic disease characteristics among those with adenocarcinoma of the endometrium receiving surgery alone or surgery followed by adjuvant RT at our institution. Despite endometrial carcinoma being one of the most common gynecologic malignancies in female tract, controversy exists regarding the role of adjuvant radiotherapy. Despite the statistically significant benefit of adjuvant RT on local control observed in the adjusted analysis, we recognize that this is a retrospective study that is prone to inherent bias. Important pathologic and clinical factors, including tumor diameter, lymphovascular space invasion, adequate lymph node evaluation, and positive peritoneal washings, were not sufficiently represented to allow for evaluation and adjustment in this series' analysis. We recognize the potential biases that these and other factors can introduce into an analysis of this kind and do not espouse these data as definitive proof of the benefit of RT in the adjuvant setting. 

 To our knowledge, this is the largest series exploring the characteristics of Mexican patients treated for endometrial carcinoma in the literature. We conclude that adjuvant radiotherapy should be considered an integral component of definitive treatment for women with unfavorable disease as a mean to decrease mortality and improve patient outcome. Future work is needed to delineate clinical and biologic factors that can guide treatment and account for disparities in outcomes between subsets of women with endometrial carcinoma, including patterns of care analyses and prospective studies of adjuvant radiotherapy modalities and tumor-specific treatment strategies.

## Figures and Tables

**Table 1:** Characteristics of Mexican patients with endometrial carcinoma.

Characteristics	Number of patients	Percentage
	412	100

*Age*		
<45	79	19.2
45−54	108	26.2
55−64	139	33.7
65−74	61	14.8
≥75	25	6.1
Mean age	55.19 (23–87)	
*Menopausal status*		
Premenopausal	61	14.8
Postmenopausal	351	85.2
*Body mass index (BMI)*		
<30 (kg/m^2^)	229	55.6
>30 (kg/m^2^)	136	33
Not registered	47	11.4
Mean BMI	28.97 (12.32–48.8)	
*Parity status*		
Nullipara	121	29.4
Multipara	291	70.6
*Blood glucose*		
<100 mg/dL	309	75.0
100 a 199 mg/dL	83	20.1
>200 mg/dL	20	4.9
Comorbid conditions		
Yes	145	35.19
No	267	64.81
Diabetes mellitus (DM)	94/412	22.81
Systemic arterial hypertension (SAH)	80/412	19.41
DM + SAH	34/412	8.25
Other comorbidities	5/412	1.21

NOS: not otherwise specified.

**Table 2:** Pathology characteristics for Mexican patients with endometrial carcinoma.

Characteristic	Patients	Percentage
*Stage FIGO 1988*		
IA	46	11.2
IB	96	23.3
IC	79	19.2
IIA	44	10.7
IIB	42	10.2
IIIA	47	11.4
IIIB	1	.2
IIIC	55	13.3
IVA	2	.5
*Grade*		
1	165	40
2	115	27.9
3	130	31.6
NOS	2	0.5
*Lymphovascular invasion*		
Yes	57	13.8
No	341	82.8
NOS	14	3.4
*Number of nodes*		
None	168	40.8
1−10	181	43.9
11– 20	51	12.4
21– 30	4	1.0
31– 40	8	1.9
Mean of nodes obtained at surgery	5 (1–40)	
*Histology*		
Endometrioid	359	87.1
Papillary serous	21	5.1
Clear cell	10	2.4
Adenosquamous	21	5.1
Glassy cell	1	.2
*Size*		
<3 cm	66	16.0
3 −5.9 cm	100	24.3
>6 cm	77	18.7
NOS	169	41.0
Mean size (cm)	4.78 (0.3–20.5)	
*Isthmus invasion*		
Present	76	18.4
Absent	336	81.6
*Uterine cervix involvement*		
Endocervical gland	52	12.6
Stromal	94	22.8
None	266	64.6
*Serous Involvement*		
Present	47	11.4
Absent	365	88.6
*Positive peritoneal washing*		
Present	16	3.9
Absent	352	85.4
NOS	44	10.7
*Adnexial involvement*		
Present	38	9.2
Absent	374	90.8
*Number of positive nodes*		
1	19/412	4.6
2	9/412	2.2
3	6/412	1.5
>3	9/412	2.2
*Myometrial invasion*		
<1/2	138	33.5
>1/2	221	53.6
None	49	11.9
NOS	4	1.0
*Para aortic node involvement*		
Present	17	4.1
Absent	395	95.9
*Pelvic node involvement*		
Present	39	9.5
Absent	373	90.5
*Risk categories*		
Low	99	24.0
Low-intermediate	55	13.3
High intermediate	47	11.4
High	211	51.2

NOS: not otherwise specified.

**Table 3:** Grade according stage in Mexican patients with endometrial carcinoma.

Stage (FIGO 1988)	Grade 1 *n* = 165 (%)	Grade 2 *n* = 115 (%)	Grade 3 *n* = 130 (%)	Not specified *n* = 2 (%)
IA	40 (24.2)	3 (2.6)	3 (2.3)	0
IB	54 (32.7)	26 (22.6)	17 (13.1)	0
IC	26 (15.7)	16 (13.9)	37 (28.5)	1 (50)
IIA	19 (11.5)	17 (14.8)	17 (13.8)	1 (50)
IIB	8 (4.8)	20 (17.4)	13 (10)	0
IIIA	11 (6.7)	17 (14.8)	18 (13.8)	1 (50)
IIIB	1 (0.7)	0	0	0
IIIC	6 (3.7)	15 (13)	33 (2.4)	0
IVA	0	1 (0.9)	1 (0.7)	0

**Table 4:** Treatment characteristics of Mexican patients with endometrial carcinoma.

Characteristic	Patients	Percentage
*Surgery *		
TAH-BSO	120	29.1
TAH-BSO-pelvic LND	20	4.9
TAH-BSO-Pelvic/para aortic LND and omentum biopsy	271	65.8
Pelvic exenteration	1	.2
*Lymph node dissection*		
Pelvic	296/412	71.8
Para aortic	273/412	66.3
*Chemotherapy*		
Yes	59	14.3
No	353	85.7
Concurrent with RT	25/59	6.1
Sequential chemotherapy	31/59	7.5
Adjuvant without RT	3/59	.7
*Chemotherapy agents used*		
Cisplatin/adriamicin	15	3.6
Cisplatin	2	.5
CisCA regimen	4	1.0
Carboplatin/adriamicin/ciclophosphamide	1	.2
PVC regimen	3	.7
Adriamicin	3	.7
Cisplatin/adriamicin/ciclophosphamide	1	.2
Ciclophosphamide/adriamicin	1	.2
Cisplatin/paclitaxel	2	.5
Carboplatin	22	5.3
Carboplatin/paclitaxel	4	1.0
Gemcitabine	1	.2
*Hormone therapy*		
Yes	10	2.4
No	402	97.6
*Radiotherapy *		
Yes	270	65.53
No	142	34.47
Pelvic EBRT	11/270	4.08
WART + pelvic RT + VBT	8/270	2.96
EBRT + VBT	241/270	89.26
VBT	7/270	2.59
Pelvic + retroperitoneum EBRT + VBT	3/270	1.11
*Brachytherapy*		
Yes	255/412	61.89
No	157/412	38.11
High-dose rate	14/255	5.49
Low-dose rate	241/255	94.51

TAH-BSO: total abdominal hysterectomy, TAH-BSO-LND: total abdominal histerectomy and lymph node dissection, EBRT: external beam radiotherapy, WART: whole abdominal radiotherapy, and VBT: vaginal vault brachytherapy.

**Table 5:** Multivariate analysis using cox regression model for prognostic factors for overall survival in patients with endometrial carcinoma.

Characteristic	HR	IC 95%	*P*
Grade			
1	1.00		
2	3.40	1.62–7.09	0.0004
3	4.97	2.44–10.10	
Serosal involvement			
Yes	2.51	1.44–4.38	0.005
No	1.00		
Adnexial involvement			
Yes	2.31	1.25–4.30	0.005
No	1.00		
Age (years)			
<45	1.00		
45–54	0.66	0.30–1.44	0.0003
55–64	0.97	0.50–1.89	
65–74	1.33	0.63–2.82	
>75	5.40	2.44–11.93	
